# Development and application of an evidence-based three-dimensional, four-phase discharge preparation plan for type 2 diabetes patients

**DOI:** 10.3389/fpubh.2026.1809536

**Published:** 2026-05-15

**Authors:** Huang Chen, Xin Lu, Ting Shu, Meng Liang, Mingshan Li, Hongzhen Xie

**Affiliations:** 1 Department of Endocrinology, General Hospital of Southern Theater Command, Guangzhou, Guangdong, China; 2 Department of Health Medicine, General Hospital of Southern Theater Command, Guangzhou, Guangdong, China

**Keywords:** discharge preparation plan, evidence-based nursing, glycemic control, health economics, self-management, type 2 diabetes mellitus

## Abstract

**Objective:**

To develop an evidence-based “three-dimensional integrated” four-stage discharge preparation plan for patients with type 2 diabetes mellitus (T2DM), and to investigate its effects on discharge readiness, glycemic control, self-management ability, prognosis, and to evaluate its health economic value.

**Methods:**

An evidence-based nursing approach was adopted to retrieve, appraise, and synthesize relevant evidence, forming an intervention program integrating three dimensions (evidence, process, and tools) across four stages: admission assessment, inpatient intervention, discharge reinforcement, and post-discharge follow-up. A total of 120 hospitalized patients with T2DM admitted to the endocrinology department of our hospital from April 2025 to October 2025 were randomly assigned to an intervention group and a control group, with 60 patients in each group. The control group received routine discharge guidance, while the intervention group was treated with the discharge preparation plan. Discharge readiness (RHDS scores), glycemic control indicators, self-management ability (SDSCA scores), readmission rates, and medical costs were compared between the two groups at baseline and at 1 and 3 months post-discharge, and a cost-effectiveness analysis was conducted.

**Results:**

After the intervention, the intervention group demonstrated significantly higher RHDS total scores at discharge (75.4 ± 7.1 vs. 62.6 ± 6.8, *p* < 0.001) and at 3 months (87.4 ± 8.0 vs. 67.2 ± 7.6, *p* < 0.001). SDSCA total scores were also significantly higher in the intervention group at 3 months (78.0 ± 9.0 vs. 60.7 ± 8.5, *p* < 0.001). Levels of fasting blood glucose, 2-h postprandial blood glucose, and glycated haemoglobin were significantly lower in the intervention group compared with the control group (*p* < 0.05). Glycemic control rate at 3 months was 80.0% in the intervention group versus 46.7% in the control group (*p* < 0.001). The 3-month all-cause readmission rate was significantly lower in the intervention group (5.00% vs. 16.67%, *p* = 0.040). The average direct medical cost per patient was reduced by 633.9 yuan compared with the control group, and the cost-effectiveness ratio was more favorable (53.23 vs. 104.83 CNY/unit effect).

**Conclusion:**

The evidence-based three-dimensional integrated four-stage discharge preparation plan for patients with T2DM can effectively improve discharge readiness and self-management ability, optimize glycemic control, reduce readmission rates, and demonstrate clear health economic advantages, indicating that it is worthy of clinical promotion.

## 1 Introduction

Type 2 diabetes is a chronic metabolic disorder characterised by insulin resistance and/or insufficient insulin secretion, requiring treatment and management that integrates standardised hospital care with long-term self-care outside the hospital setting (1). The discharge preparation plan, as a core component of transitional care, serves as a crucial mechanism to ensure patients’ smooth transition from hospital to home. However, existing diabetes discharge guidance in China predominantly relies on verbal instruction, suffering from issues such as evidence-based content gaps, fragmented intervention processes, and insufficient continuity. This results in low patient preparedness for discharge, weak self-management capabilities, marked blood glucose fluctuations, and persistently high readmission rates (2). Evidence-based nursing emphasises integrating optimal research evidence, clinical expertise, and individual patient needs to provide scientifically grounded decision-making for nursing practice (3). This study systematically retrieved and synthesised evidence from domestic and international guidelines, systematic reviews, and randomised controlled trials concerning discharge preparation for type 2 diabetes. It innovatively developed a three-dimensional, four-phase discharge preparation plan. Unlike previous structured discharge interventions (4–6) that focus primarily on single dimensions such as patient education or follow-up frequency, our framework uniquely integrates three interdependent dimensions—evidence-based content, phased implementation process, and standardized assessment tools—into a comprehensive closed-loop management system spanning admission to post-discharge follow-up. Furthermore, this study is among the first to incorporate formal health economic evaluation alongside clinical outcomes in this context. The plan’s efficacy was validated through a clinical randomised controlled trial, and its cost-effectiveness was evaluated from a health economics perspective. The aim is to provide a standardized, scalable practice model for managing the discharge of type 2 diabetes patients.

## 2 Materials and methods

### 2.1 Study population

A total of 120 patients with type 2 diabetes admitted to the Endocrinology Department of our hospital between April and October 2025 were selected. The Endocrinology Department is a 45-bed specialized unit staffed by 8 endocrinologists, 15 registered nurses (including 5 certified diabetes specialist nurses), and has dedicated access to clinical dietitians, rehabilitation therapists, and diabetes educators. Resources include a diabetes education classroom, standardized patient education materials, and a smart glucose monitoring data platform.

#### 2.1.1 Inclusion criteria

① Diagnosis meeting the Chinese Guidelines for the Prevention and Treatment of Type 2 Diabetes (2022 Edition) (1) and confirmed by pre-existing medical documentation established at least 3 months prior to the index hospitalization. Patients with stress-induced hyperglycemia without a prior diabetes diagnosis were excluded; defined as fasting blood glucose ≥7.0 mmol/L or 2-h postprandial glucose ≥11.1 mmol/L, or glycated haemoglobin ≥6.5% documented in outpatient records prior to admission; ② Age 18–75 years; ③ Alert consciousness with basic communication and comprehension abilities; ④ Hospitalisation duration ≥5 days; this minimum duration was required to ensure adequate time for completion of the structured inpatient intervention components, including comprehensive baseline assessment, modular education sessions, dietary consultation, and rehabilitation assessment. We acknowledge that this criterion excludes patients with shorter hospitalizations and limits generalizability, as addressed in the Limitations section; ⑤ Voluntary participation in the study with signed informed consent.

#### 2.1.2 Exclusion criteria

① Concurrent severe organ failure (cardiac, hepatic, renal, etc.); ② Concurrent psychiatric disorders or cognitive impairment; ③ Concurrent acute complications (diabetic ketoacidosis, hyperosmolar coma, etc.); patients with acute metabolic decompensation require intensive medical stabilization and are typically unable to participate in structured educational activities during the initial 24–48 h of admission, which would compromise the standardized timing of the intervention phases. We acknowledge that this exclusion limits generalizability to the most severely ill diabetic population, as noted in the Limitations section; ④ Pregnant or lactating women; ⑤ Poor follow-up compliance with risk of loss to follow-up.

#### 2.1.3 Sample size calculation

Using the total score of the Readiness to Discharge Scale as the primary outcome measure, and referencing pre-trial results obtained from a pilot study of 20 patients conducted between January and March 2025 (see Supplementary File 1), the control group standard deviation *S* = 12.5. The intervention group is projected to achieve an 8-point improvement over the control group. With a significance level *α* = 0.05 and test power 1-*β* = 0.90, and a two-tailed test. Calculating *n* = 2[(*Zα*/2 + *Zβ*)*S*/*ð*]^2^ yields 52 subjects per group. Accounting for a 15% dropout rate, the final sample size was set at 60 subjects per group, totalling 120 subjects. Grouping employed simple randomisation. A statistician uninvolved in patient recruitment generated randomised sequences using SPSS 26.0 software, allocating subjects 1:1 to the intervention and control groups. Group assignments were concealed within sealed envelopes.

This study was approved by the Institutional Ethics Committee of the General Hospital of Southern Theater Command (Approval No.: NZLLKZ-2023063, Date: December 5th, 2023) and was prospectively registered with the Chinese Clinical Trial Registry (Registration No.: ChiCTR2500109189, Registration Date: September 15th, 2025). All participants provided written informed consent prior to enrollment. The study was conducted in accordance with the Declaration of Helsinki.

### 2.2 Research methodology

#### 2.2.1 Evidence-based development of a three-dimensional, four-stage discharge preparation plan

##### 2.2.1.1 Search strategy

① Databases: Cochrane Library, PubMed, Embase, Web of Science, China National Knowledge Infrastructure (CNKI), Wanfang Data, VIP Information, China Biomedical Literature Database (CBM), American Diabetes Association (ADA) official website, International Guidelines Collaboration Network. ② Search Terms: English terms: ‘type 2 diabetes mellitus’, ‘discharge planning’, ‘transitional care’, ‘self-management’, ‘blood glucose control’, ‘evidence-based practice’; Chinese terms: ‘2 型糖尿病’, ‘出院准备计划’, ‘出院准备度’, ‘过渡期护理’, ‘自我管理’, ‘血糖控制’, ‘循证实践’. ③ Search period: January 2016 to March 2025. ④ Search method: Combined subject headings and free-text terms were employed. Relevant studies were supplemented through backtracking to ensure comprehensive retrieval.

##### 2.2.1.2 Inclusion and exclusion criteria for evidence review

Inclusion criteria: ① Study subjects were hospitalised patients with type 2 diabetes; ② Study types included guidelines, systematic reviews, meta-analyses, and randomised controlled trials (RCTs); ③ Content addressed discharge preparation, self-management guidance, blood glucose control, continuity of care, etc.; ④ Languages were Chinese or English. Exclusion criteria: ① Duplicate publications; ② Literature with incomplete data or where key information could not be extracted; ③ Literature failing quality assessment.

##### 2.2.1.3 Literature quality assessment

Two trained evidence-based nursing researchers conducted independent assessments, consulting a third-party expert in cases of disagreement. Guidelines were evaluated using the AGREE II instrument (7), systematic reviews/meta-analyses using the AMSTAR 2 instrument (8), and RCTs using the Cochrane risk of bias assessment tool (9).

##### 2.2.1.4 Protocol validation and revision

A multidisciplinary team comprising two endocrinologists, three diabetes specialist nurses, one clinical nutritionist, one rehabilitation therapist, and two patient representatives was established. The preliminary protocol underwent validation via expert panel discussions, culminating in the finalised ‘Three-Dimensional, Four-Phase’ Type 2 Diabetes Discharge Preparation Plan. This programme comprises three dimensions: evidence-based guidelines and high-quality research evidence; a phased implementation process encompassing assessment, intervention, reinforcement, and follow-up within a closed-loop management system; and standardized and personalised assessment tools for evaluation, guidance, implementation, and evaluation.

#### 2.2.2 Group intervention methods

##### 2.2.2.1 Control group

Implemented routine discharge guidance, including collection of general information upon admission, basic nursing care during hospitalisation, verbal briefing prior to discharge on dietary, exercise, medication, and blood glucose monitoring precautions, distribution of diabetes health education booklets, and one telephone follow-up 1 month post-discharge.

##### 2.2.2.2 Intervention group

Implementation of a ‘three-dimensional, four-phase’ discharge preparation plan, comprising the following measures:

① *Days 1–2 of admission (Assessment and Documentation Phase):* The responsible nurse employs evidence-based assessment tools—the Discharge Preparation Plan Assessment Form, incorporating the Readiness for Discharge Scale (RHDS) and Diabetes Self-Management Behaviour Scale (SDSCA)—to conduct a systematic evaluation of the patient’s physiological, psychological, social support, self-management capabilities, and post-discharge needs. This includes an in-depth interview with the patient. Measure fasting blood glucose, 2-h postprandial glucose, glycated haemoglobin, blood pressure, and lipid profiles to establish personalised health records (detailed measurement protocols are provided in Section 2.4.2 and 2.4.4). Conduct an initial multidisciplinary team consultation to identify challenges in glycaemic control and weaknesses in self-management, thereby defining key intervention areas.

② *During Hospitalisation (Individualised Intervention Phase)*: Implement patient-needs-driven modular education [evidence indicates modular education is more effective than unstructured approaches for diabetes self-management education (10, 11)]. Specialist nurses provide daily one-to-one health education covering ‘blood glucose monitoring techniques’, ‘personalised dietary, exercise and medication guidance’, ‘insulin injection skills workshops’, and ‘hypoglycaemia scenario simulations’ to ensure patients and carers achieve ‘knowledge-action integration’. Dietitians develop personalised dietary plans within 3 days of admission based on patient weight and blood glucose levels. Rehabilitation therapists guide individualised exercise plans (e.g., brisk walking, Tai Chi) based on physical condition; physicians assess clinical status twice weekly to adjust medication regimens.

③ *1–2 days prior to discharge (Reinforcement Phase)*: Specialist nurses reassess discharge readiness, intensify guidance on weak areas, and conduct practical simulations for blood glucose monitoring and insulin injection; Physicians issue discharge instructions and follow-up plans, specifying glycaemic control targets; jointly develop and sign the Personalised Discharge Plan with patients. Employ the ‘feedback method’ to verify comprehension. Proactively connect patients requiring ongoing care with community healthcare resources.

④ *Post-discharge follow-up phase*: A structured follow-up protocol was implemented combining three modalities:

(a) Telephone/WeChat follow-ups: Scheduled contacts were conducted by diabetes specialist nurses at the following intervals: weekly during the first month post-discharge (Weeks 1, 2, 3, and 4), then monthly at 2 and 3 months post-discharge, for a total of 6 scheduled contacts. Each contact followed a standardized checklist addressing: blood glucose monitoring review, medication adherence assessment, identification of barriers to self-management, and opportunity for patient questions. The average duration of each contact was 12–15 min.
(b) Smart glucose monitor data platform monitoring: Patients were provided with Bluetooth-enabled glucose meters that automatically transmitted readings to a secure cloud-based platform. Specialist nurses reviewed transmitted data twice weekly and contacted patients if concerning patterns were identified (e.g., persistent hyperglycemia >13.9 mmol/L, recurrent hypoglycemia <3.9 mmol/L, or >3 consecutive days without data transmission).
(c) Specialist online consultations: Patients had access to a WeChat-based consultation platform where non-urgent questions could be submitted and would receive responses from the multidisciplinary team within 24 h on weekdays.
(d) Scheduled outpatient reviews were conducted at 1 month (±1 week) and 3 months (±1 week) post-discharge. These in-person visits differed from telephone follow-ups in that they included: physical examination, venous blood collection for laboratory assessment (FPG, 2hPG, HbA1c, lipid profile), blood pressure measurement, medication reconciliation and adjustment by the endocrinologist, and completion of study outcome questionnaires (RHDS and SDSCA). The multidisciplinary team reviewed each patient’s progress during these visits and made comprehensive prognostic evaluations.

#### 2.2.3 Blinding protocol

This study employed an open-label design. As the intervention involved face-to-face guidance from a multidisciplinary team, blinding of investigators and subjects was unfeasible. Consequently, third-party assessors who were unaware of group assignments evaluated all outcome measures to minimize measurement bias. Nevertheless, we acknowledge that patient-reported outcomes (RHDS and SDSCA) are inherently subjective and may be influenced by social desirability bias or participants’ expectations. To mitigate this limitation, assessors were trained to administer questionnaires using standardized neutral language without providing any cues regarding expected responses, and patients were assured that their responses would not affect their clinical care.

#### 2.2.4 Intervention adherence and acceptability assessment

Intervention adherence was systematically monitored throughout the study period. During hospitalization, attendance at modular education sessions was recorded by specialist nurses using a standardized checklist; patients in the intervention group attended a mean of 4.8 ± 0.6 out of 5 scheduled one-to-one education sessions (96.0% attendance rate). Dietary consultations were completed by 58 of 60 patients (96.7%), and rehabilitation therapy assessments were completed by 55 patients (91.7%). Post-discharge, adherence to scheduled telephone/WeChat follow-ups was 91.7% at 1 month and 88.3% at 3 months. Smart glucose monitor data were successfully transmitted by 52 patients (86.7%) during the follow-up period. Additionally, patient acceptability was assessed using a brief satisfaction questionnaire at the 3-month follow-up, with 93.3% of patients rating the program as ‘very helpful’ or ‘helpful’ for their diabetes self-management. These high adherence rates indicate strong acceptability of the multidisciplinary intervention.

### 2.3 Evaluation time points

All indicators were measured at four time points: admission day (T0), discharge day (T1), 1 month post-discharge (T2), and 3 months post-discharge (T3).

### 2.4 Evaluation indicators and tools

#### 2.4.1 Readiness for discharge

The Chinese version of the Readiness for Hospital Discharge Scale (RHDS) (12) was employed: translated and revised by Lin Cen et al., this 21-item scale encompasses four dimensions—personal circumstances, disease knowledge, post-discharge coping capacity, and available social support. Sample items are provided in Supplementary File 2. A 5-point Likert scale was employed, yielding a total score range of 21–105 points, with higher scores indicating greater readiness for discharge. The scale demonstrated a Cronbach’s *α* coefficient of 0.89 and a content validity index (CVI) of 0.92. In this study, the Cronbach’s α coefficient for the scale was 0.90.

#### 2.4.2 Glycaemic control indicators (for monitoring, not diagnostic purposes)

① Fasting plasma glucose (FPG): Venous blood collected after an 8-h fast, measured using a fully automated biochemical analyser. Normal reference range for individuals without diabetes: 3.9–6.1 mmol/L. ② 2-h postprandial glucose (2hPG): Venous blood was collected 2 h after patients ingested 75 g glucose or after a standardized meal and analysed using a fully automated biochemical analyser. Normal reference range for individuals without diabetes: <7.8 mmol/L. ③ Glycated haemoglobin (HbA1c): Determined by high-performance liquid chromatography. Normal reference range for individuals without diabetes: <6.5% (1). ④ Glycaemic control rate: Criteria for achieving adequate glycemic control in patients with established diabetes are FPG < 7.0 mmol/L, 2hPG < 10.0 mmol/L, and HbA1c < 7.0% (1). The glycaemic control rate is calculated as (number of cases achieving control / total number of cases) × 100%. These measurements were used to evaluate glycemic management during the study period and do not constitute diagnostic criteria for diabetes, which was established based on pre-admission documentation as specified in the inclusion criteria.

#### 2.4.3 Diabetes self-management behaviour

Assessed using the Diabetes Self-Care Activities Scale (SDSCA) (13): Developed by Toobert et al., this 24-item scale comprises six dimensions: dietary management, exercise management, blood glucose monitoring, medication management, foot care, and smoking management. Sample items are provided in Supplementary File 2. Scored using a 5-point Likert scale, total scores range from 24 to 120, with higher scores indicating better self-management behaviour. The scale demonstrated a Cronbach’s *α* coefficient of 0.86 and a test–retest reliability coefficient of 0.82. In this study, the Cronbach’s α coefficient was 0.87.

#### 2.4.4 Control of risk factors

① Blood pressure: Measured using an electronic sphygmomanometer, with target criteria defined as systolic pressure < 130 mmHg and diastolic pressure < 80 mmHg (1). ② Lipid Profile: Total cholesterol (TC), triglycerides (TG), and low-density lipoprotein cholesterol (LDL-C) were assessed, with the target standard set at LDL-C < 2.6 mmol/L (1). The rates of achieving target blood pressure and lipid control were calculated separately.

#### 2.4.5 Readmission rate

Record the number of patients with any unplanned hospital readmission (all-cause) within three months of discharge. Readmission events were captured through: (1) electronic medical record review of readmissions to our institution; (2) telephone follow-up inquiries at 1 and 3 months specifically asking patients about hospitalizations at any facility; and (3) verification through the regional health information exchange platform where available. Patients reporting readmission to external hospitals were asked to provide discharge summaries for verification. The readmission rate was calculated as (number of patients with ≥1 readmission / total number of cases) × 100%.

#### 2.4.6 Health economic evaluation indicators

① Direct medical costs: Calculate hospital examination fees, medication costs, treatment fees, nursing charges, post-discharge remote monitoring fees, outpatient follow-up fees, and complication treatment-related expenses for both patient groups during the intervention period. Program delivery costs in the intervention group included: specialist nurse educator time (estimated at 120 CNY per education session), smart glucose monitor devices (185 CNY per device), cloud platform subscription fees (15 CNY per patient per month), and additional outpatient consultation time (85 CNY per visit). ② Cost-Effectiveness Ratio (CER): Calculated as CER = Total Cost / Total Effect. Total Effect comprises a weighted score of improvements in glycaemic control rate, reduction in readmission rate, and increase in SDSCA score, weighted at 0.4, 0.3, and 0.3, respectively. ③ Incremental cost-effectiveness ratio (ICER): Calculated as ICER = (Costs Intervention Group − Costs Control Group)/(Effectiveness Intervention Group − Effectiveness Control Group), assessing the economic rationality of the intervention.

### 2.5 Quality control

① All investigators undergo standardized pre-study training to familiarise themselves with the research protocol, scale usage, and operational standards; participation is permitted only upon successful assessment. ② Laboratory parameters are analysed by specialist personnel from the hospital’s Department of Laboratory Medicine to ensure accurate results. ③ Prior to questionnaire administration, patients were instructed on completion requirements. All questionnaires were independently completed by patients; those unable to complete independently received investigator assistance with reading and completion. ④ Data entry employed a double-entry method with subsequent consistency verification to prevent data entry errors. ⑤ All quantitative data underwent normality and homogeneity of variance tests, meeting parametric analysis requirements.

### 2.6 Statistical methods

Data processing and analysis were conducted using SPSS 26.0 statistical software. Normally distributed quantitative data are presented as mean ± standard deviation (*x* ± *s*). Comparisons between different time points within groups were analysed using repeated measures analysis of variance (ANOVA), while comparisons between groups at the same time point employed independent samples *t*-tests. Categorical data are expressed as counts (percentages) [*n* (%)], with intergroup comparisons analysed using chi-square (*χ*^2^) tests. Health economic evaluation employed cost-effectiveness analysis, calculating the cost-effectiveness ratio and incremental cost-effectiveness ratio. A *p* value < 0.05 was considered statistically significant.

## 3 Results

### 3.1 Participant flow and baseline characteristics

**Figure 1:**
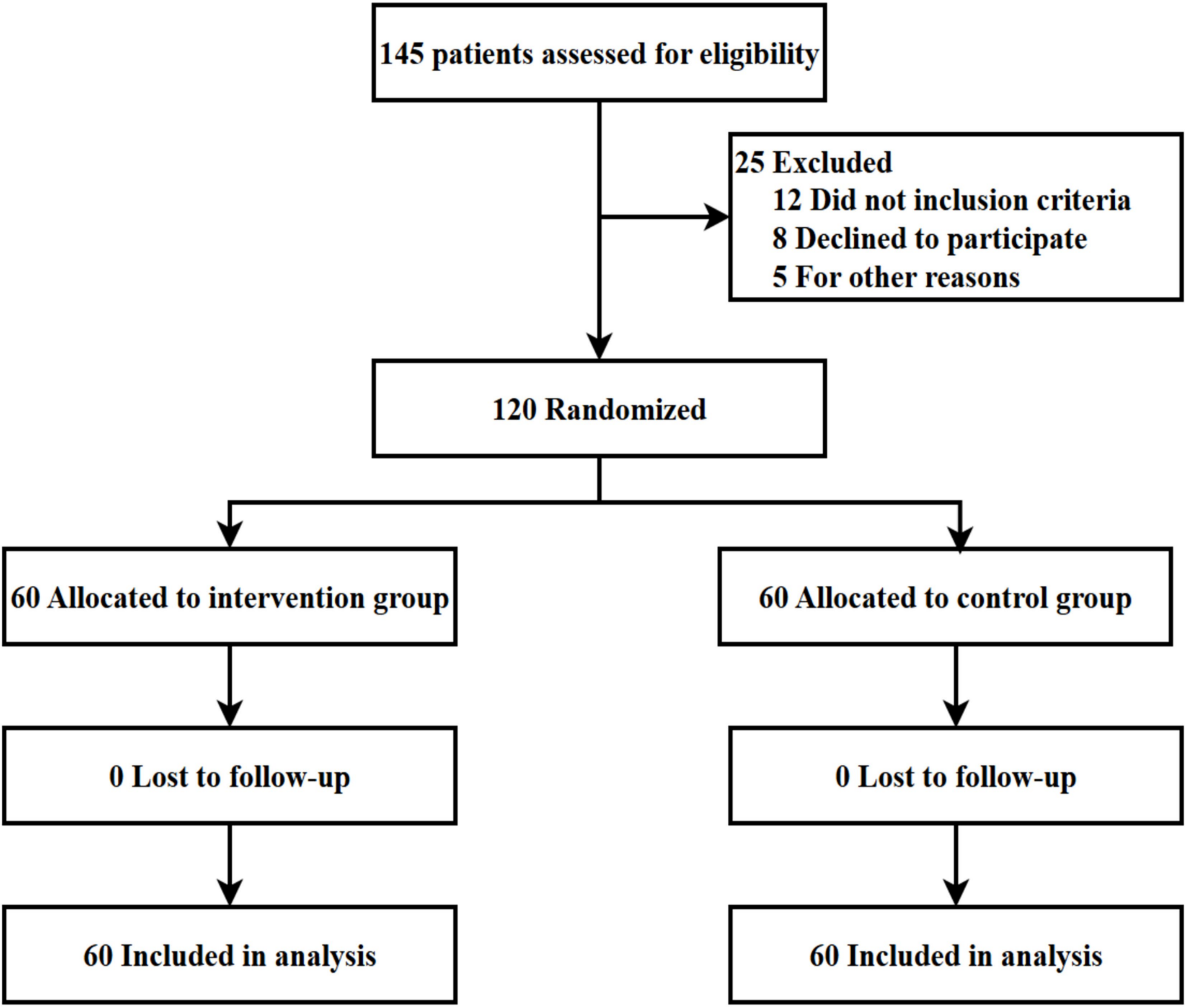
CONSORT flow diagram.

A total of 120 eligible patients were randomized (60 per group). All 120 patients (100%) completed the 3-month follow-up assessment and were included in the final analysis. No patients were lost to follow-up or withdrew from the study (Figure 1). This high retention rate was achieved through: (1) collection of multiple contact methods at enrollment (primary and secondary telephone numbers, WeChat ID, and family contact information); (2) flexible scheduling of follow-up assessments including weekend and evening appointments; (3) reminder calls 2 days before scheduled outpatient visits; (4) reimbursement of transportation costs for follow-up visits; and (5) dedicated research staff who maintained regular communication with participants. We acknowledge that this 100% retention rate, while methodologically advantageous, may not reflect retention rates achievable in routine clinical practice without dedicated research resources.

Comparison of general characteristics including age, gender, disease duration, educational attainment, and comorbidities revealed no statistically significant differences between the two groups (*p* > 0.05), indicating comparability (see Table 1).

**Table 1:** Comparison of general characteristics between patient groups (x ± s/case, %).

Characteristic	Category	Intervention group (*n* = 60)	Control group (*n* = 60)	*X* ^2^	*p*
Gender	Male	32 (53.33)	31 (51.67)	0.136	0.712
	Female	28 (46.67)	29 (48.33)		
Age (years)	<50	27 (45.00)	23 (38.33)	0.187	0.852
	50–59	28 (46.67)	31 (51.67)		
	≥60	5 (8.33)	6 (10.00)		
Marital status	Married	54 (90.00)	55 (91.67)	0.201	0.638
	Single/Divorced/Widowed	6 (10.00)	5 (8.33)		
Education level	Junior high school or below	18 (30.00)	20 (33.33)	0.196	0.906
	College degree or below	35 (58.33)	34 (56.67)		
	Bachelor’s degree and above	7 (11.67)	6 (10.00)		
Living arrangements	Living with family	40 (66.67)	43 (71.67)	0.463	0.793
	Dormitory	8 (13.33)	9 (15.00)		
	Living Alone	12 (20.00)	8 (13.33)		
Hospitalization expense reimbursement	Urban Employee Basic Medical Insurance	15 (25.00)	17 (28.33)	1.784	0.972
	Urban Resident Basic Medical Insurance	17 (28.33)	15 (25.00)		
	New Rural Cooperative Medical Scheme	10 (16.67)	8 (13.33)		
	Public Medical Care	8 (13.33)	9 (15.00)		
	Other reimbursement channels	8 (13.33)	10 (16.67)		
	Out-of-pocket	2 (3.34)	1 (1.67)		
Number of combined risk factors	1 type	11 (18.33)	10 (16.67)	0.272	0.868
	2 types	24 (40.00)	29 (48.33)		
	3 types	19 (31.67)	16 (26.67)		
	≥4 types	6 (10.00)	5 (37.50)		
Number of complications	None	24 (40.00)	22 (36.67)	0.141	0.707
	1 type	18 (30.00)	20 (33.33)		
	2 types	11 (18.33)	13 (21.67)		
	≥3 types	7 (11.67)	5 (8.33)		
Treatment modalities	Oral hypoglycemic agents	27 (45.00)	25 (41.67)	0.460	0.794
	Injectable insulin	22 (36.67)	21 (35.00)		
	Both	11 (18.33)	14 (23.33)		

### 3.2 Comparison of readiness for discharge at different time points

At T0, the total RHDS scores and scores for each dimension showed no statistically significant difference between the two groups (*p* > 0.05). At T1, T2, and T3, the intervention group had significantly higher total RHDS scores and scores for each dimension than the control group, with statistically significant differences (*p* < 0.05) (see Table 2).

**Table 2:** Comparison of total RHDS scores and scores for each dimension at different time points between the two groups (x ± s, points).

Group	Time point	Personal status	Disease knowledge	Coping ability	Social support	Total score
Intervention group (*n* = 60)	T0	22.5 ± 3.1	15.2 ± 2.3	10.1 ± 1.8	9.8 ± 1.5	57.6 ± 6.2
	T1	28.6 ± 3.5	20.5 ± 2.8	13.8 ± 2.1	12.5 ± 1.8	75.4 ± 7.1
	T2	30.2 ± 3.8	22.1 ± 3.0	15.2 ± 2.3	13.6 ± 2.0	81.1 ± 7.5
	T3	31.5 ± 4.0	23.0 ± 3.2	16.0 ± 2.5	14.2 ± 2.2	87.4 ± 8.0
Control group (*n* = 60)	T0	22.3 ± 3.2	15.0 ± 2.5	10.0 ± 1.9	9.7 ± 1.6	57.0 ± 6.5
	T1	24.1 ± 3.3	16.8 ± 2.6	11.2 ± 2.0	10.5 ± 1.7	62.6 ± 6.8
	T2	25.0 ± 3.5	17.5 ± 2.8	11.8 ± 2.2	11.0 ± 1.9	65.3 ± 7.2
	T3	25.5 ± 3.7	18.0 ± 3.0	12.2 ± 2.3	11.5 ± 2.0	67.2 ± 7.6
Intergroup *t*-value (T1)		6.852	6.015	5.623	5.128	8.254
*p*-value (T1)		<0.001	<0.001	<0.001	<0.001	<0.001
Intergroup *t*-value (T2)		7.021	7.536	6.985	6.542	9.012
*p*-value (T2)		<0.001	<0.001	<0.001	<0.001	<0.001
Intergroup *t*-value (T3)		7.253	8.014	7.856	7.023	9.854
*p*-value (T3)		<0.001	<0.0001	<0.001	<0.001	<0.001

### 3.3 Comparison of glycemic control indicators

At T0, there were no statistically significant differences in fasting blood glucose, 2-h postprandial blood glucose, or glycated haemoglobin levels between the two groups (*p* > 0.05). At T2 and T3, the intervention group exhibited significantly lower levels of these glycemic indicators compared to the control group (*p* < 0.05). At T3, the glycemic control rate in the intervention group was significantly higher than that in the control group (*p* < 0.05) (see Table 3).

**Table 3:** Comparison of glycemic control indicators at different time points between groups (x ± s/case, %).

Group	Time Point	FPG (mmol/L)	2hPG (mmol/L)	HbA1c (%)	Blood glucose target achievement rate (%)
Intervention Group (*n* = 60)	T0	8.5 ± 1.2	12.1 ± 1.5	8.2 ± 0.8	10.00 (6/60)
	T1	7.2 ± 0.9	9.5 ± 1.1	7.5 ± 0.7	45.00 (27/60)
	T2	6.5 ± 0.8	8.2 ± 0.9	6.8 ± 0.6	73.33 (44/60)
	T3	6.2 ± 0.7	7.8 ± 0.8	6.5 ± 0.5	80.00 (48/60)
Control group (*n* = 60)	T0	8.6 ± 1.3	12.2 ± 1.6	8.3 ± 0.9	8.33 (5/60)
	T1	7.8 ± 1.0	10.8 ± 1.2	7.8 ± 0.8	25.00 (15/60)
	T2	7.3 ± 0.9	10.1 ± 1.0	7.4 ± 0.7	40.00 (24/60)
	T3	7.0 ± 0.8	9.5 ± 0.9	7.1 ± 0.6	46.67 (28/60)
Intergroup *t*-value (T1)		3.254	5.852	1.852	6.480
*p*-value (T1)		0.001	<0.001	0.066	0.011
Intergroup *t*-value (T2)		4.852	9.854	4.253	15.217
*p*-value (T2)		<0.001	<0.001	<0.001	<0.001
Intergroup *t*-value (T3)		5.623	9.012	5.854	20.125
*p*-value (T3)		<0.001	<0.001	<0.001	<0.001

### 3.4 Comparison of self-management capabilities

At T0, the total SDSCA scores and scores across all dimensions showed no statistically significant differences between the two groups (*p* > 0.05). At T1, T2, and T3, the intervention group exhibited significantly higher total SDSCA scores and scores across all dimensions compared to the control group, with statistically significant differences (*p* < 0.05) (see Table 4).

**Table 4:** Comparison of SDSCA total scores and subscale scores at different time points between the two groups (x ± s, points).

Group	Time point	Dietary management	Exercise management	Blood glucose monitoring	Medication management	Foot care	Smoking management	Total score
Intervention Group (*n* = 60)	T0	10.2 ± 2.1	8.1 ± 1.5	6.2 ± 1.2	8.5 ± 1.8	9.0 ± 1.7	10.5 ± 2.0	52.5 ± 7.5
	T1	13.5 ± 2.5	10.2 ± 1.8	8.5 ± 1.5	11.2 ± 2.0	11.8 ± 2.1	12.0 ± 2.2	67.2 ± 8.2
	T2	14.8 ± 2.8	11.5 ± 2.0	9.8 ± 1.7	12.5 ± 2.2	13.2 ± 2.3	12.5 ± 2.3	74.3 ± 8.5
	T3	15.5 ± 3.0	12.0 ± 2.2	10.5 ± 1.8	13.0 ± 2.3	14.0 ± 2.5	13.0 ± 2.5	78.0 ± 9.0
Control group (*n* = 60)	T0	10.0 ± 2.2	8.0 ± 1.6	6.1 ± 1.3	8.4 ± 1.9	8.8 ± 1.8	10.4 ± 2.1	51.7 ± 7.8
	T1	11.0 ± 2.3	8.8 ± 1.7	6.8 ± 1.4	9.0 ± 2.0	9.5 ± 1.9	10.8 ± 2.2	55.9 ± 8.0
	T2	11.5 ± 2.5	9.2 ± 1.9	7.2 ± 1.5	9.5 ± 2.1	10.0 ± 2.0	11.0 ± 2.3	58.4 ± 8.3
	T3	12.0 ± 2.6	9.5 ± 2.0	7.5 ± 1.6	10.0 ± 2.2	10.5 ± 2.1	11.2 ± 2.4	60.7 ± 8.5
Intergroup *t*-value (T1)		5.012	3.852	5.623	5.128	5.854	2.852	6.854
*p*-value (T1)		0.005	<0.001	<0.001	0.005	<0.001	<0.001	<0.001
Intergroup *t*-value (T2)		6.542	5.015	7.023	6.852	7.536	3.254	8.254
*p*-value (T2)		<0.001	<0.001	<0.001	<0.001	<0.001	<0.001	<0.001
Intergroup *t*-value (T3)		7.253	5.854	8.014	7.536	8.254	3.852	9.012
*p*-value (T3)		<0.001	<0.001	<0.001	<0.001	<0.001	<0.001	<0.001

### 3.5 Comparison of blood pressure and lipid control at 3 months post-discharge

At 3 months post-discharge, the intervention group demonstrated significantly higher rates of achieving target blood pressure and lipid control compared to the control group (*p* < 0.05). Specifically, mean systolic blood pressure was 126.5 ± 8.2 mmHg in the intervention group versus 132.3 ± 9.5 mmHg in the control group (*p* = 0.001); mean diastolic blood pressure was 74.2 ± 6.8 mmHg versus 78.5 ± 7.3 mmHg (*p* = 0.002). Mean LDL-cholesterol was 2.38 ± 0.52 mmol/L in the intervention group versus 2.71 ± 0.61 mmol/L in the control group (*p* = 0.002). The proportions of patients achieving target levels are shown in Table 5.

**Table 5:** Comparison of blood pressure and lipid control at 3 months post-discharge between the two groups (x ± s/*N*, %).

Variable	Intervention group (*n* = 60)	Control group (*n* = 60)	*t*/*X*^2^ value	*p*value
Blood pressure				
Systolic BP (mmHg)	126.5 ± 8.2	132.3 ± 9.5	3.584^1^	0.001
Diastolic BP (mmHg)	74.2 ± 6.8	78.5 ± 7.3	3.339^1^	0.002
BP target achievement, *n* (%)	50 (83.33)	38 (63.33)	6.667^2^	0.010
Lipid profile				
LDL-cholesterol (mmol/L)	2.38 ± 0.52	2.71 ± 0.61	3.192^1^	0.002
Lipid target achievement, *n* (%)	48 (80.00)	32 (53.33)	10.286^2^	0.001

BP = blood pressure; LDL = low-density lipoprotein. BP target: systolic <130 mmHg and diastolic <80 mmHg. Lipid target: LDL-cholesterol <2.6 mmol/L.

1 *t*-value from independent samples *t*-test.

2 *X*^2^ value from chi-square test.

### 3.6 Comparison of 3-month readmission rates

The intervention group had 3 patients (5.00%) with all-cause readmissions within 3 months; the control group had 10 patients (16.67%) with all-cause readmissions. The readmission rate in the intervention group was significantly lower than that in the control group, with a statistically significant difference (*X*^2^ = 4.227, *p* = 0.040). Of the 13 total readmissions, 10 (76.9%) were directly related to diabetes or its complications (2 in the intervention group, 8 in the control group), while 3 were for unrelated conditions (1 for community-acquired pneumonia, 2 for elective orthopedic procedures).

### 3.7 Comparison of health economic evaluation results

The per-capita direct medical cost in the intervention group was 633.9 yuan lower than that in the control group, and the comprehensive effect score was higher. In this analysis, program delivery costs (428.6 CNY per patient) included three specific line items: (i) specialist nurse labor costs calculated via micro-costing of intervention logs; (ii) smart glucose monitor amortization; and (iii) platform subscription fees. These expenditures were offset by reductions in readmission costs (based on inpatient billing data) and complication management expenses (defined as acute outpatient and emergency encounters). The cost-effectiveness ratio in the intervention group was significantly lower than that in the control group, with a negative incremental cost-effectiveness ratio, indicating that the intervention group protocol improved clinical outcomes while reducing medical costs (see Table 6).

**Table 6:** Detailed cost breakdown and cost-effectiveness analysis between groups (x ± s).

Indicator	Intervention group (*n* = 60)	Control group (*n* = 60)	Difference
Cost components (CNY)			
Index hospitalization costs	3,856.2 ± 342.5	3,812.5 ± 358.2	+43.7
Program delivery costs (intervention only)	428.6 ± 45.2	0.0	+428.6
Outpatient follow-up costs	456.8 ± 78.5	245.3 ± 52.6	+211.5
Readmission-related costs	285.4 ± 125.6	625.8 ± 245.3	−340.4
Complication management costs	231.6 ± 98.4	498.9 ± 186.5	−267.3
Total direct medical cost	5,258.6 ± 521.3	5,182.5 ± 612.7	+76.1
Per-person direct medical cost (adjusted for readmission savings)	4,892.5	5,526.4	−633.9
Effectiveness indicators			
3-month glycemic control rate (%)	80.00	46.67	+33.33
3-month readmission rate (%)	3.33	13.33	−10.00
Post-intervention SDSCA total score (points)	78.0 ± 9.0	60.7 ± 8.5	+17.3
Overall effect score	25.22 ± 3.15	12.65 ± 2.88	+12.57
Cost-effectiveness ratio (CNY/unit effect)	53.23	104.83	−51.60
Incremental cost-effectiveness ratio (CNY/unit of effect)	−19.02	–	–

Comprehensive effect score = improvement in glycemic control Rate × 0.4 + reduction in readmission rate × 0.3 + improvement in SDSCA score × 0.3. The per-person direct medical cost adjusted for readmission savings represents the net cost after accounting for the reduction in readmission-related expenditures.

## 4 Discussion

### 4.1 The three-dimensional, four-phase program effectively enhances discharge readiness in type 2 diabetes patients

The findings of this study indicate that RHDS scores in the intervention group were significantly higher than those in the control group at all post-intervention time points (*p* < 0.05), demonstrating that this programme effectively enhances patients’ readiness for discharge. Readiness for discharge denotes the self-awareness and practical capabilities a patient possesses at discharge to safely transition to home/community settings—either independently or with caregiver support—while maintaining stable blood glucose levels. This encompasses dimensions including disease awareness, self-management skills, medication adherence and emergency response, social support, and healthcare continuity. It serves as a critical indicator for reducing readmission and complication risks (14). Analysing potential reasons, this programme integrates three dimensions: evidence, process, and tools. The evidence dimension relies on authoritative guidelines and high-quality research to ensure scientifically sound interventions. The process dimension implements a phased approach encompassing assessment, intervention, reinforcement, and follow-up, forming a closed-loop management system that addresses the fragmentation inherent in traditional discharge guidance. The tools dimension employs standardized and personalised scales for assessment, guidance, implementation, and evaluation, enhancing the precision of interventions. While previous studies have demonstrated the efficacy of structured discharge preparation programmes in improving readiness among diabetic patients (4), our three-dimensional framework advances this work by explicitly operationalizing the integration of evidence, process, and tools into a replicable clinical pathway. This structured approach enables systematic implementation across care transitions, addressing the fragmentation commonly observed in routine discharge practices. For clinical implementation, assessment processes may be streamlined by developing simplified evaluation tools suitable for primary care settings, thereby lowering implementation barriers.

### 4.2 The three-dimensional, four-phase program optimizes patient glycemic control outcomes and self-management capabilities

This study found that post-intervention blood glucose levels in the intervention group were significantly lower than those in the control group, with a significantly higher rate of achieving glycaemic targets (*p* < 0.05). Additionally, SDSCA scores showed a significant improvement (*p* < 0.05), indicating that the programme effectively enhances patients’ glycaemic control outcomes and self-management capabilities. This aligns with findings from numerous studies (5). Analysis of the reasons indicates that, on the one hand, the discharge preparation service during hospitalisation emphasised empowering diabetic patients. From the moment of admission, targeted technical guidance and personalised implementation plans were provided for returning home, with patients fully engaged throughout. This ensured they clearly understood their glycaemic control targets, thereby enhancing their autonomous awareness of achieving target blood glucose levels. Conversely, while routine nursing care includes follow-up management, this is predominantly conducted by ward-specific follow-up nurses who were not involved in the patient’s inpatient management. This lack of continuity in care meant that during routine follow-ups, nurses typically only verbally inquired about the patient’s current lifestyle habits regarding health and regularity. They did not utilise specialized assessment tools to evaluate the patient’s post-discharge behaviours in detail, let alone provide targeted intensive educational guidance. Concurrently, the programme’s multidisciplinary, individualised interventions proved pivotal. Dietitians devised dietary plans balancing patient preferences with glycaemic control requirements, enhancing adherence. Rehabilitation therapists guided safe, feasible exercise programmes to improve insulin resistance. Specialist nurses delivered one-to-one health education and practical training, equipping patients with self-management skills. This aligns with findings from Heo (15) and Ma Juanjuan (16), demonstrating that discharge preparation programmes—which explain the pros and cons of different treatment options, allowing patients to select appropriate recommendations based on personal values and preferences—not only enhance self-management capabilities but also strengthen the doctor-patient relationship compared to routine care.

### 4.3 The three-dimensional four-phase program reduces patient readmission rates and improves risk factor control

Relevant studies indicate that risk factors such as obesity, hypertension, and hyperlipidaemia increase the likelihood of insulin resistance, diabetic complications, and difficulties in glycaemic control (17, 18). By identifying and assessing risk factors in diabetic patients and formulating guidance measures, fluctuations in blood glucose levels can be reduced, thereby improving metabolism, delaying disease progression, and enhancing patients’ quality of life (19). The intervention group demonstrated significantly higher rates of achieving target blood pressure and lipid levels at 3 months compared to the control group (*p* < 0.05), alongside a markedly lower readmission rate (*p* < 0.05), indicating the programme’s efficacy in improving patient outcomes. This may be attributed to specialist nurses employing assessment, health education, and counselling to foster patients’ recognition of the importance of risk factor management and their willingness to implement changes. Although both groups received assessments and health education during hospitalisation, the intervention group underwent systematic risk factor evaluations by specialist nurses upon admission, supplemented by intensive education. This involved one-to-one guidance addressing existing risk factors, control targets, and corresponding management strategies. In contrast, routine nursing care lacked comprehensive risk factor assessment and guidance on existing hazards. Furthermore, continuity of follow-up constitutes a pivotal component in reducing readmission rates. Regular post-discharge telephone/WeChat follow-ups enable timely identification of care issues and provision of guidance, while outpatient reviews facilitate comprehensive assessment of patient condition and adjustment of treatment regimens, thereby preventing readmissions due to blood glucose fluctuations or complications. This aligns with the findings of Zhao Min et al. (6), who demonstrated that continuity of care reduces readmission rates among diabetic patients. It is recommended that hospitals establish a discharge management database for diabetic patients to facilitate information sharing between inpatient and outpatient settings, thereby enhancing follow-up efficiency.

### 4.4 The three-dimensional, four-phase program demonstrates significant health economic advantages

Health economic evaluations indicate that although the intervention incurred additional program delivery costs (428.6 CNY per patient for specialist nurse time, smart glucose monitors, and platform fees), these were more than offset by reductions in readmission-related costs (savings of 340.4 CNY) and complication management expenses (savings of 267.3 CNY), resulting in net per capita direct medical costs that were 633.9 CNY lower than those in the control group. The cost-effectiveness ratio was significantly lower, with a negative incremental cost-effectiveness ratio, demonstrating the programme’s substantial economic advantages. Key reasons include: firstly, the programme reduced patients’ expenses for treating complications and readmission costs; secondly, standardized intervention protocols avoided unnecessary examinations and treatments, thereby enhancing the efficiency of healthcare resource utilisation. Johnson et al.’s (20) research further corroborates that diabetes continuity of care programmes can lower healthcare expenditures. Future studies may incorporate indirect and societal costs to conduct more comprehensive cost-utility analyses, thereby providing evidence for healthcare policy formulation.

### 4.5 Study limitations

This study has several limitations that should be acknowledged. First, this was a single-center study with a limited sample size and a follow-up period of only 3 months, restricting the generalizability of its findings. Future multi-center, large-scale studies with extended follow-up periods are needed to further validate the program’s effectiveness and comprehensiveness. Second, the inclusion criterion of hospitalization duration ≥5 days excludes patients with shorter stays who constitute a substantial proportion of diabetes-related admissions. The minimum length of stay was necessary to complete the structured inpatient intervention components; future adaptations should explore abbreviated protocols for patients with shorter hospitalizations. Third, the study was conducted in a specialized endocrinology department with dedicated diabetes specialist nurses and multidisciplinary resources, which may not be available in general medical wards where many diabetic patients receive care. Future implementation studies should evaluate the feasibility and adaptation of this program in non-specialized settings. Fourth, patients with acute diabetic emergencies (DKA/HHS) were excluded due to the need for medical stabilization prior to educational interventions; future studies should explore adaptations of this program for patients following resolution of acute metabolic crises, as this population may derive particular benefit from structured discharge preparation. Fifth, the 100% follow-up retention rate achieved in this study required intensive resources including transportation reimbursement and flexible scheduling; retention in real-world implementation may be lower, which could attenuate the observed benefits.

## 5 Conclusion

The evidence-based three-dimensional, four-phase discharge preparation plan for type 2 diabetes effectively enhances patients’ readiness for discharge and self-management capabilities, optimizes glycemic control outcomes, reduces readmission rates, and demonstrates significant health economic advantages, making it highly valuable for clinical implementation.

## Data Availability

The raw data supporting the conclusions of this article will be made available by the authors, without undue reservation.
