# Supplementary material for: Development and application of an evidence-based three-dimensional, four-phase discharge preparation plan for type 2 diabetes patients
Source: Front Public Health. 2026 May 15;14:1809536. doi: 10.3389/fpubh.2026.1809536 (PMC13219262; doi:10.3389/fpubh.2026.1809536)
Supplement: Supplementary file 1 [file Table_1.DOCX]

**Supplementary Material 1: Pre-experiment Data of Sample Size Calculation**

**Pilot Study Data (January-March 2025, n=20)**

|  | Intervention Group (n=10) | Control Group (n=10) |
| --- | --- | --- |
| RHDS Total Score at Discharge (Mean ± SD) | 74.2±11.8 | 66.3±13.1 |
| Mean Difference | 7.9 points |  |
| Pooled Standard Deviation | 12.5 |  |

Based on these pilot data, the sample size calculation assumed a control group SD of 12.5 and an expected improvement of 8 points in the intervention group, consistent with the observed pilot difference of 7.9 points.
