# Supplementary material for: Development and application of an evidence-based three-dimensional, four-phase discharge preparation plan for type 2 diabetes patients
Source: Front Public Health. 2026 May 15;14:1809536. doi: 10.3389/fpubh.2026.1809536 (PMC13219262; doi:10.3389/fpubh.2026.1809536)
Supplement: Supplementary file 2 [file Table_2.DOCX]

**Supplementary Material 2: Simplified Version of RHDS and SDSCA Scales (Sample Items)**

RHDS Sample Items (21 items total, 5-point Likert scale: 1=Strongly Disagree to 5=Strongly Agree)

Personal Status Dimension (7 items)

· I feel physically ready to go home.

· I have enough energy to take care of myself at home.

Disease Knowledge Dimension (8 items)

· I understand what symptoms to monitor after discharge.

· I know when to seek medical help if problems arise.

Coping Ability Dimension (4 items)

· I feel confident I can manage my care at home.

· I know who to contact if I have questions.

Social Support Dimension (2 items)

· I have family/friends who can help me after discharge.

· I have access to transportation for follow-up appointments.

**SDSCA Sample Items (24 items total, asks about number of days in past week: 0-7)**

Dietary Management (5 items)

· On how many of the last SEVEN DAYS did you follow a healthful eating plan?

·On how many of the last SEVEN DAYS did you eat five or more servings of fruits and vegetables?

Exercise Management (3 items)

· On how many of the last SEVEN DAYS did you participate in at least 30 minutes of physical activity?

Blood Glucose Monitoring (4 items)

· On how many of the last SEVEN DAYS did you test your blood sugar?

Medication Management (3 items)

· On how many of the last SEVEN DAYS did you take your recommended diabetes medications?

Foot Care (5 items)

· On how many of the last SEVEN DAYS did you check your feet?

Smoking Management (4 items)

· Have you smoked a cigarette—even one puff—during the past SEVEN DAYS? (Yes/No, reverse scored)
